# Does lower water availability limit stem CO_2_ efflux of oak and hornbeam coppices?

**DOI:** 10.1093/aobpla/plae023

**Published:** 2024-04-05

**Authors:** Eva Darenova, Robert Knott, Tomáš Vichta

**Affiliations:** Global Change Research Institute of the Czech Academy of Sciences, Belidla 986/4a, 60300 Brno, Czech Republic; Department of Forest Ecology, Faculty of Forestry and Wood Technology, Mendel University in Brno, Zemedelska 1665/1, Brno 613 00, Czech Republic; Department of Silviculture, Faculty of Forestry and Wood Technology, Mendel University in Brno, Zemedelska 1665/1, Brno 613 00, Czech Republic; Department of Geology and Soil Science, Faculty of Forestry and Wood Technology, Mendel University in Brno, Zemedelska 1665/1, Brno 613 00, Czech Republic

**Keywords:** *Carpinus betulus*, *Quercus petraea*, soil water content, stem growth, stem respiration, summer drought

## Abstract

Recent changes in water availability can be crucial for the development, growth and carbon budget of forests. Therefore, our aim was to determine the effect of reduced throughfall and severe summer drought on stem CO_2_ efflux as a function of temperature and stem increment. Stem CO_2_ efflux was measured using the chamber method on oak and hornbeam under four treatments: coppice, thinned coppice, and both coppice and thinned coppice with 30 %-reduced throughfall. The first year of the experiment had favourable soil water availability and the second year was characterized by a dry summer. While reduced throughfall had no effect on stem CO_2_ efflux, the summer drought decreased efflux by 43–81 % during July and August. The stem CO_2_ efflux was reduced less severely (by 13–40 %) in September when the drought persisted but the stem increment was already negligible. The stem increment was also strongly affected by the drought, which was reflected in its paired relationship with stem CO_2_ efflux over the two experimental years. The study showed that summer dry periods significantly and rapidly reduce stem CO_2_ efflux, whereas a constant 30 % rainfall reduction needs probably a longer time to affect stem properties, and indirectly stem CO_2_ efflux.

## Teaser

Recent changes in water availability can be crucial for the development, growth and carbon budget of forests. We investigated the response of stem growth and stem CO_2_ efflux of oak and hornbeam coppices to a 2-year constant mild soil water reduction and temporal severe drought. The mild soil water reduction was enabled by drainage channels preventing 30 % of throughfall from reaching the ground and did not have any significant impact on stem growth or CO_2_ efflux. On the contrary, the severe drought decreased stem CO_2_ efflux by 43–81 % during July and August as a result of reduced stem growth. In September, when the drought persisted but the stem increment was already negligible, stem CO_2_ efflux was affected less severely (by 13–40 %). The study showed that summer dry periods significantly and rapidly reduce stem CO_2_ efflux and stem growth.

## Introduction

The increasing severity and frequency of drought as a consequence of global climate change are expected to affect tree functioning (Wolf and Paul-Limoges 2023). Drought can be understood as low soil water availability or as high atmospheric water deficit, but these usually co-occur (Zhou *et al.* 2019). Droughts have a substantial impact on carbon fluxes within forest ecosystems, as they can affect gross primary production, carbon allocation, growth (Rambal *et al.* 2014; Krejza *et al.* 2022) and respiration of soil and plants (Schindlbacher *et al.* 2012; Rodríguez-Calcerrada *et al.* 2014). Drought also alters the carbon sink-source ratio (Wolf and Paul-Limoges 2023). According to a recent study (Xiao *et al.* 2021) on different forest ecosystems worldwide, drought often has a bigger impact on carbon assimilation than on respiration. Moreover, Jardine *et al.* (2022) found that growth processes are suppressed by the drought faster than photosynthesis. Forests absorb and store less CO_2_ from the atmosphere, which substantially hinders their role in climate change mitigation.

Respiration is the process used by trees to obtain the energy necessary for the maintenance of existing structures and the production of new tissues (Amthor 2000). Through autotrophic respiration, trees release around 30–50 % of the carbon assimilated through photosynthesis (gross primary production) (De Lucia *et al.* 2007; Fernández-martínez *et al.* 2014; Kunert *et al.* 2019; West 2020). Therefore, it is an important carbon flux. Tree stems account for a substantial part of carbon allocation in the ecosystem, ranging between 25 % and 80 % of net ecosystem productivity (Babst *et al.* 2014), which is important for both forest growth and economic purposes. Stem respiration accounts for an estimated 7–25 % of the total respiration of mature forest ecosystems (Malhi *et al.* 1999; Zha *et al.* 2004; Acosta *et al.* 2008; Khomik *et al.* 2010; Yang *et al.* 2016). Stem respiration is especially driven by changes in temperature (Darenova *et al.* 2018a) and by energy demands for stem growth (Darenova *et al.* 2020) which includes cambium activation, dividing and elongation of cells and cell wall thickening (Gruber *et al.* 2009). The respiration connected with the stem growth can contribute by up to 55 % to the annual total stem respiration (Lavigne and Ryan 1997; Zha *et al.* 2004; Rodríguez-calcerrada *et al.* 2019; Darenova *et al.* 2020) Stem growth is sensitive to climate change and reduced stem growth due to drought has been described by Krejza *et al.* (2022), Van Kampen *et al.* (2022) and Liu *et al.* (2023). Although an increase in stem respiration of drought-treated tropical trees has been observed (e.g. Rowland *et al.* 2018), a decrease in stem respiration has been observed during dry periods in other studies (Rodríguez-Calcerrada *et al.* 2014, 2021; Salomón *et al.* 2018). This decrease can be explained by a drought-induced reduction in stem growth and, thus, a decrease in growth respiration. Dry conditions can also alter the effect of the factors on stem respiration. According to Saveyn *et al.* (2007a), stem CO_2_ efflux under well-watered conditions followed the diurnal changes in temperature, while it was synchronized with diurnal variations in stem diameter during drought. Along with the intensity, the drought timing during the year is important in determining the extent to which drought affects stem growth (Krejza *et al.* 2022).

In areas with frequent or extreme drought periods (e.g. Mediterranean areas or areas affected by climate change; Logli and Joffre 2001), coppice could be considered as one of the suitable options for forest management (Pietras *et al.* 2016). Coppicing is a form of traditional forest management that utilizes how vegetative sprouts of broadleaf tree species emerge from stumps or roots shortly after the main tree has been cut (Evans 1992). Due to rapid resprouting and regeneration, it was used mainly for firewood production. With the utilization of new energy sources, coppicing decreased in the 20th century (Sieferle 2001). The higher drought resistance of the coppice compared to seedlings in their early development stages comes from their root system already being established from the previous generation or rotations and allowing for greater access to water and nutrients (Herrero *et al.* 2014). Nevertheless, the resistance of a coppice stand can be also affected by silvicultural practices. For example, forest thinning can increase throughfall, reduce competition between individual trees, and promote the growth and water use of remaining trees (Rodríguez-Calcerrada *et al.* 2011; del Campo *et al.* 2022).

We carried out an experiment focussing on the effect of a 30 %-throughfall reduction, simulating mild drought conditions, on stem CO_2_ efflux (Rs) and stem growth on two tree species eight to nine years after coppicing. Measurements of Rs were carried out over 2 years, one with favourable SWC and one with a dry summer. Thus, we could compare the effect of a continuous mild drought to a temporal severe drought. Moreover, we investigated whether thinning results in any improvements for the drought resistance of the studied processes.

Our aims were to determine Rs as a function of temperature and stem increment under both mentioned soil water availability conditions. We hypothesized that: (i) continuous mild drought simulated by 30 %-throughfall reduction would not affect Rs while severe drought would reduce it; (ii) the decrease in Rs due to the drought would be a function of reduction of stem increment; and (iii) thinning would reduce the impact of severe drought on sprouts.

## Material and Methods

### The study site and experimental design

The study plots were situated in the Training Forest Enterprise Masaryk Forest Krtiny, Bilovice forest district, in the south-eastern part of the Czech Republic (49°25ʹ N, 16°68ʹ E). The site was located at an altitude of 323 m a.s.l. on a western slope with a 5–10° inclination and is characterized by a mean annual temperature of 7.5 °C and mean annual precipitation of 550–650 mm. The soil is classified as Cambisol on the granodiorite base with an admixture of Devonian and Loess sediments in the upper part of the soil profile.

The original forest was a 45-year-old stand dominated by sessile oak (*Quercus petraea* [Matt.] Liebl.). In 2008, a coppice stand was established here after a clear-cut on an area of 40 × 125 m following the methodology of Kadavý *et al.* (2011). The tree species in the experimental coppice stand are sessile oak (73.3 % of total stand basal area), European hornbeam (*Carpinus betulus*; 15.5 % of total stand basal area) and silver birch (*Betula pendula*; 15.5 % of total stand basal area) and European beech (*Fagus sylvatica*) (0.7 % of total stand basal area).

The coppiced area was divided into four rectangles (area of 20 × 31 metres). In two of the rectangles, thinning was performed with a hand saw at the ground level in the winter 2014/2015. The basal area of each stool (multi-stemmed tree) was reduced to 50 % by leaving 1–3 dominant sprouts per stump for sessile oaks and 1–5 dominant sprouts per stump for European hornbeam. The thinning reduced the stand density from 17,953 to 8021 and from 16,424 to 8021 sprouts per hectare on the plot without and with reduced throughfall, respectively. On two plots (one unthinned and one thinned), parallel drainage channels were installed about 50 cm above the ground to reduce throughfall. The drainage channels were made of wood with the surface covered by plastic strips and had a V-shape of a width of 20 cm. They covered 30 % of the plot surface and drained the rainwater outside the coppiced area.

In summary, four plots with a different combination of treatments were studied: C-0, coppice stand; T-0, thinned coppice stand; C-r coppice stand with reduced throughfall; and T-r, thinned coppice stand with reduced throughfall. Twelve sprouts from six oak and six hornbeam individual stools per treatment were chosen for ecophysiological measurements. All were stump-resprouters and were 8 years of age at the end of 2016. Their average height was 4.8 m for oak and 4.9 m for hornbeam, which corresponded to 116 % and 118 % of the average height of all trees, respectively. The average diameter of the sampled trees was 6.1 m for oak and 4.8 cm for hornbeam, which corresponded to 155 % and 121 % of the average diameter of all trees, respectively. Selected trees were a minimum of 5 m from the plot edge to avoid the border effect and were from the upper canopy layer so as to be classified as dominant. We assumed that no heartwood was developed in the sprouts yet, following the findings of Sousa *et al.* (2013) for another *Quercus* species that starts to form heartwood when the stem diameter reaches about 10–20 cm.

### Stem CO_2_ efflux

Stem respiration was estimated as CO_2_ efflux from a stem segment during six and four campaigns during the growing seasons of 2016 and 2017, respectively. The length of the measured segments ranged between 50 and 80 mm and the stem diameter of the segments ranged between 46.9 (±5.9) and 57.4 (±5.9) mm for oak and between 35.7 (±6.1) and 47.1 (±8.4) mm for hornbeam [see Supporting Information Fig. S1]. The measurements were performed between 9 a.m. and 1 p.m. in the same order of plots: C-0, T-0, T-r and C-r. We used a closed dynamic (non-steady-state, through-flow) chamber technique. We used custom-made cylindrical opaque plastic chambers [see Supporting Information Fig. S2] adapted for different stem diameters. The chamber consisted of two identical parts between which the sprout was placed, then the chamber was tightened with a strap. The adjacent edges of the chamber parts were made using neoprene sealing and any leakages between the chamber and the sprout were filled with a reusable putty-like pressure-sensitive adhesive. The chamber was only fixed on a sprout (80–100 cm above the ground) during CO_2_ efflux measurement and connected to the CO_2_ analyser LI-8100 (LI-COR, USA). LI-8100 recorded CO_2_ concentration in the chamber in 1-s intervals for one minute. A linear approach was used to calculate stem CO_2_ efflux per stem surface (R_S_; µmol m^–3^s^–1^) according to the equation:


$$\begin{array}{l} R_{S}=\frac{P\cdot V_{1}\cdot \partial c}{R\cdot T\cdot V_{2}\cdot \partial t}, \end{array}$$

where *P* is the air pressure (Pa), *V*_1_ is the volume of the system (m^3^), *R* is the molar gas constant, *T* is the sample air temperature (K), *V*_2_ is the volume of the sprout segment (m^3^) and δ*c*/δ*t* is the change in CO_2_ concentration in time. During each campaign, *V*_2_ was calculated for each sprout from two orthogonal diameters (measured with digital callipers) in the middle of the sprout segment closed in the chamber.

The natural diurnal stem temperature fluctuation affects Rs. Since the measurement campaign took about 4 h, we could not eliminate a potential increase in stem temperature from plot to plot, which can bias evaluating differences in Rs between the plots. Therefore, we normalized Rs to the temperature of 10 °C to determine the treatment effect on *R*_*s*_ regardless of different temperatures. We used an equation:


$$\begin{array}{l} R_{15}=\frac{R_{S}}{Q_{10}^{T_{s}-15/10}}, \end{array}$$

where R_15_ is stem CO_2_ efflux at 15 °C, *T*_*s*_ is the stem temperature and *Q*_10_ is the proportional change in *R*_*s*_ in relation to a 10 °C increase in temperature. *Q*_10_ was set to be two according to the mean *Q*_10_ for oak species summarized in Salomón *et al.* (2017).

### Stem growth and tree water deficit

Changes in diameter were recorded in 1-h intervals using automated band dendrometers EMStrap 21 and DRL 26, which use the same type of sensor with identical accuracy (manufactured by EMS Brno, Czech Republic). They were fixed on the sprouts 50 cm above the ground as *R*_*s*_ was performed. In such detailed time series, tree stem physiology processes of irreversible growth and reversible swelling and shrinkage are recorded (Zweifel *et al.* 2021). For this reason, we used the ‘zero growth’ approach (Zweifel *et al.* 2016, 2021) to partition original stem diameter changes into periods with and without growth to get the accumulative growth time series (irreversible increasing of the stem). Diameter variations below the preceding maximum stem diameter were considered as periods of tree water deficit (TWD), a proxy for stem dehydration and tree drought (Zweifel *et al.* 2016).To determine seasonal courses of daily increment rate (µm day^−1^) and daily TWD maxima (µm), we calculated the 7-day moving averages.

Due to technical problems, we missed measurements on stem growth at the beginning of the growing season 2017 (until 13 July) on the C-r plot.

### Micrometeorological measurements

Stem surface temperature was measured during each Rs measurement using a thermocouple probe HH12B (OMEGA Engineering Inc., CZ).

Soil water content (SWC) at 25 cm was measured in 1-h intervals using a VIRRIB sensor (Litschmann 1991). Due to a malfunction of the SWC probe, we missed data before 10 July 2016. However, stem CO_2_ efflux was measured that year on the same days as soil CO_2_ efflux (Darenova *et al.* 2018b) accompanied by SWC measurements at a depth of 0–6 cm (ThetaProbe ML2x, Delta-T Devices, Cambridge, UK). Therefore, we used a linear fit between these two SWCs separately for each treatment to estimate SWC at 25 cm during the first three Rs measurement campaigns in 2016. The coefficients of determinations of these linear fits were 1.00 for C-0, 0.91 for T-0, 0.84 for C-r and 0.94 for T-r.

Precipitation (rain gauge Pronamic Pro in conjunction with event recorder Minikin ERi; EMS Brno, Czech Republic), air temperature (*T*_*a*_) and humidity (RH) (Minikin RTHi; EMS Brno, Czech Republic) were recorded continuously in 1-h intervals. Vapour pressure deficit (VPD, hPa) was calculated according to the following equation (Allen *et al.* 1998):


$$\begin{array}{l} VPD=\left(0.6108\cdot e^{\frac{17.2694\cdot Ta}{237.3+Ta}}\right)\cdot \left(1-\frac{RH}{100}\right) \end{array}$$

### Statistical analyses

A mixed model was used to assess the overall effect of throughfall reduction, thinning and their combination on R_15_ and stem increment for oak and hornbeam. Tree individuals and the day of measurement were chosen as random effects. The final version of the model form can be written as follows:


$$\begin{array}{l} R_{15}\;or\;\;Increment\;\;∼\;1+Thinning\;+\;Reduction+Thinning\cdot \;Reduction\;+\;(1\;|\;Tree)+(1\;|\;Measurement\;\;day) \end{array}$$

Differences in mean SWC from measurement days between the treatments were tested by ANOVA on repeated measures. The differences in R_15_ and stem increments between tree species and individual plots in individual measurement days were tested using Two-way ANOVA on repeated measures. The dependence of the variability in R_15_ on sprout diameter and 7-day average stem diameter increment was tested using the Pearson correlation test during each measurement campaign. The effect of the mean increment rate on the mean R_15_ over two growing seasons and the difference in this effect between oak and hornbeam was tested with analysis of covariance (ANCOVA). To compare Rs in 2016 and 2017, we determined linear regressions between Rs and stem temperature (means from each campaign) in 2016 (a year without drought). Then, we calculated the Rs residuals for 2017 measurements from these regressions. Due to the little effect of the treatments tested with the Mixed model, we grouped the data from all plots together. The statistical significance of the analyses was tested at the *α* = 0.05 probability level. The statistical analyses were performed using SigmaPlot 11.0 analytical software (Systat Software, San Jose, CA, USA). The mixed model was performed in the R-studio environment using the lme4 package (version 1.1-26), and estimates of P values using the lmerTest package (version 3.1-3).

## Results

### Micrometeorology

Precipitation for the period between April and September amounted to 340 and 389 mm in 2016 and 2017, respectively. Air humidity was the lowest between April and August. Air humidity was lower and VPD was substantially higher in the summer of 2017 compared to 2016 [see Supporting Information Fig. S3] despite the similar seasonal precipitation. According to long-term climatic data from the nearest meteorological station Turany Brno (CHMI 2024), temperatures in both years were above average of the climate normal while the summer precipitation and air humidity in 2017 were substantially lower [see Supporting Information Fig. S4]. This confirms that the experimental forest was exposed to dry conditions.

**Figure 1.**
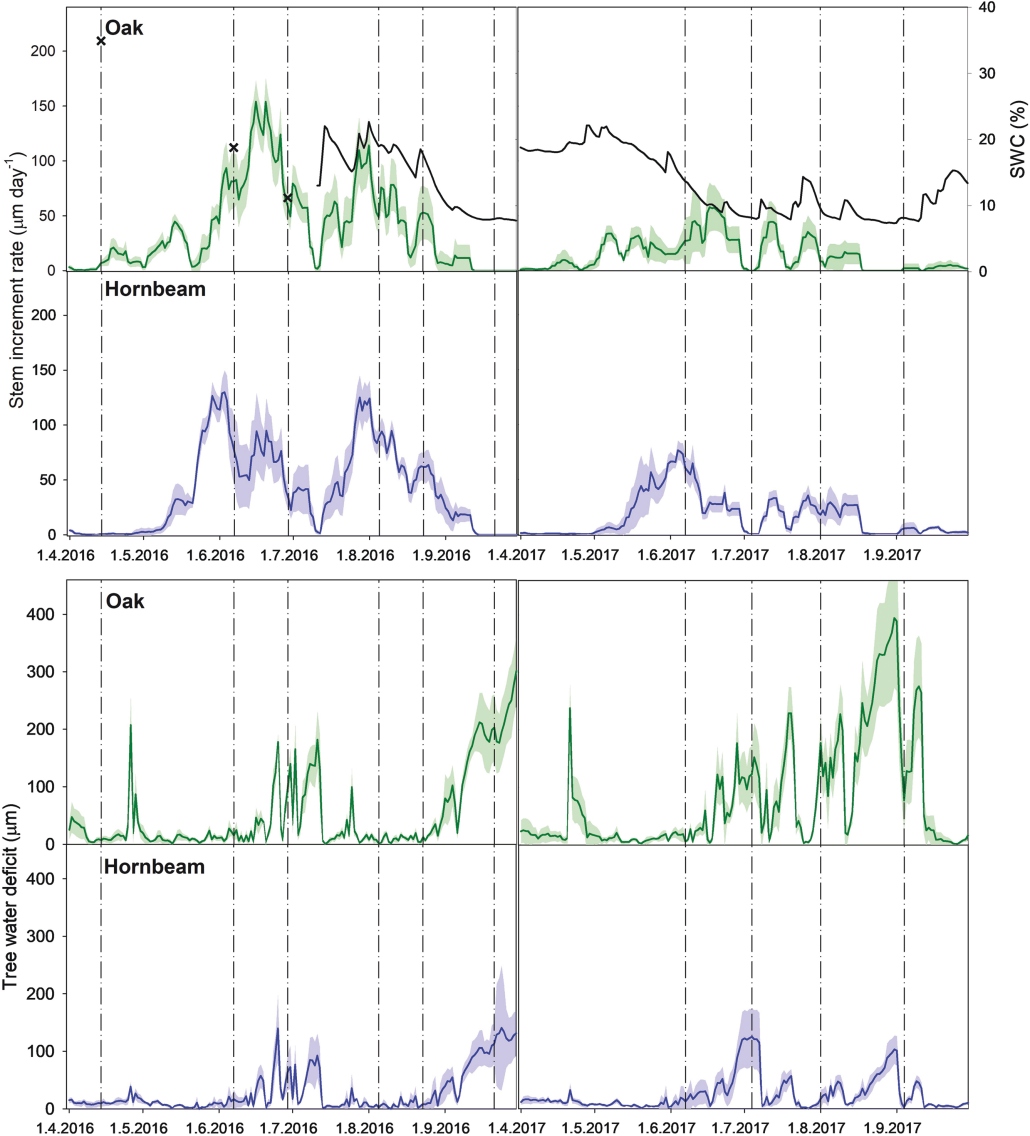
Seven-day moving averages of the daily stem diameter growth rate and TWD maxima calculated from six oak and six hornbeam sprouts, and daily mean soil water content (SWC; black line) at 25 cm depth on the control coppice plot over two growing seasons. The coloured shadings represent the standard deviation resulting from the six repetitions per tree species. The dot-dashed lines show days of stem CO_2_ efflux measurements. The crosses are SWC estimated according to manual measurements.

**Figure 2.**
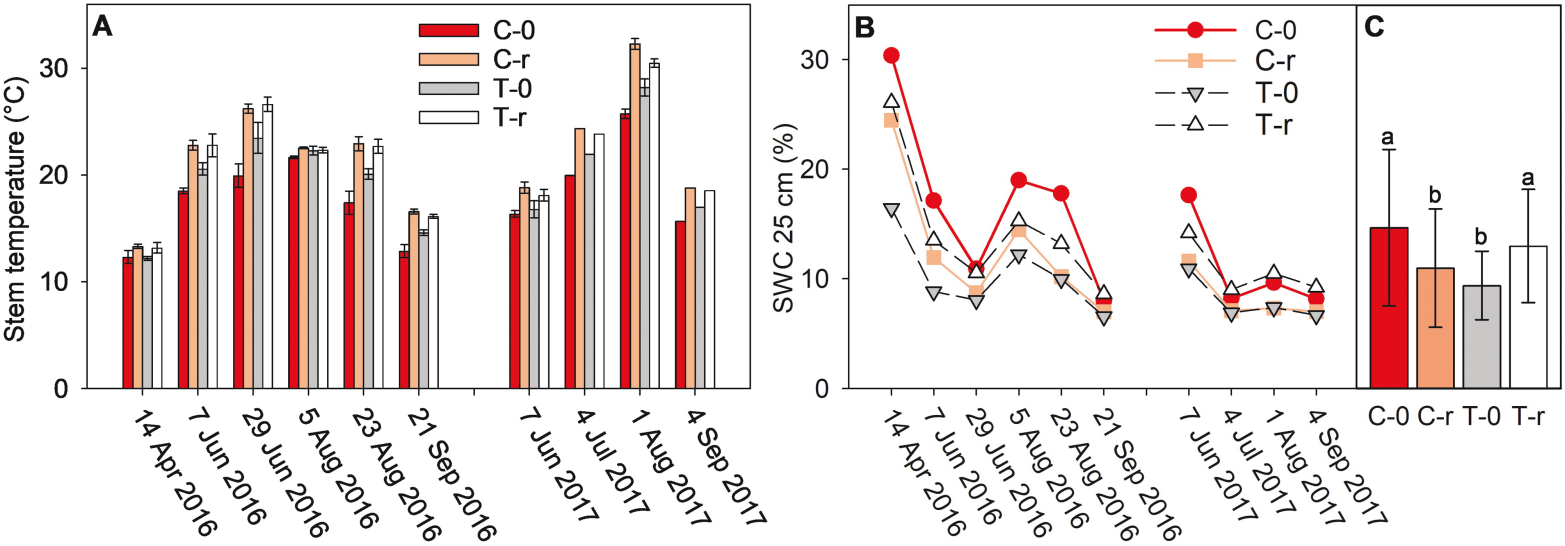
Micrometeorological conditions during Rs measurement campaigns. A—mean (± SD) stem temperature from 10 to 12 sprouts per plot; B—SWC at a depth of 25 cm; C—mean (± SD) SWC from campaign days over two seasons. Four plots with different treatments were studied: C-0, a coppice stand; T-0, a thinned coppice stand; C-r, a coppice stand with reduced throughfall and T-r, thinned coppice stand with reduced throughfall. Different letters in the C indicate significant differences between the treatments (*P* < 0.05; one-way ANOVA on repeated measures).

SWC in the control plot usually ranged between 10 % and 22 % in 2016 (Fig. 1). At the beginning of the experimental season of 2017, SWC was around 20%. However, it gradually started to decrease in May, and from June to August SWC fluctuated mostly below 10 %. On the measurement campaign days, SWC in C-0 and T-r were statistically higher than in C-r and T-0 (Fig. 2C). Low SWC and high VPD in the summer of 2017 confirm the severity of the drought affecting the experimental stand.

Stem temperature during Rs measurements ranged between 12 °C and 33 °C with a maximum on 1 August 2017 (Fig. 2A). The temperatures followed the pattern of C-0 < T-0 < T-r < C-r, which corresponds to the timing of measurements.

### Stem growth and TWD

Generally, the sprouts grew faster in 2016 compared to 2017 (Fig. 1). The annual stem diameter increment of oak was 7.12 (± 1.5) mm and 2.81 (± 1.11) mm in 2016 and 2017, respectively, and that of hornbeam was 7.44 (± 1.26) mm and 3.11 (± 0.75) mm. The cumulative stem increment of the two species during the 2 years is shown in Supporting Information Fig. S5.

There were two peaks of the stem diameter increment rate during one growing season. The first peak occurred at the edge of May and June for hornbeam and in mid-June for oak. Daily increments during this first peak reached rates around 130 µm day^–1^ for hornbeam and around 150 µm day^–1^ for oak in 2016. The peaks were about half the size in 2017. The second peak was at the end of August with similar rates (around 120 µm day^–1^) for both tree species. In 2017, the second peak was less distinct.

**Figure 3.**
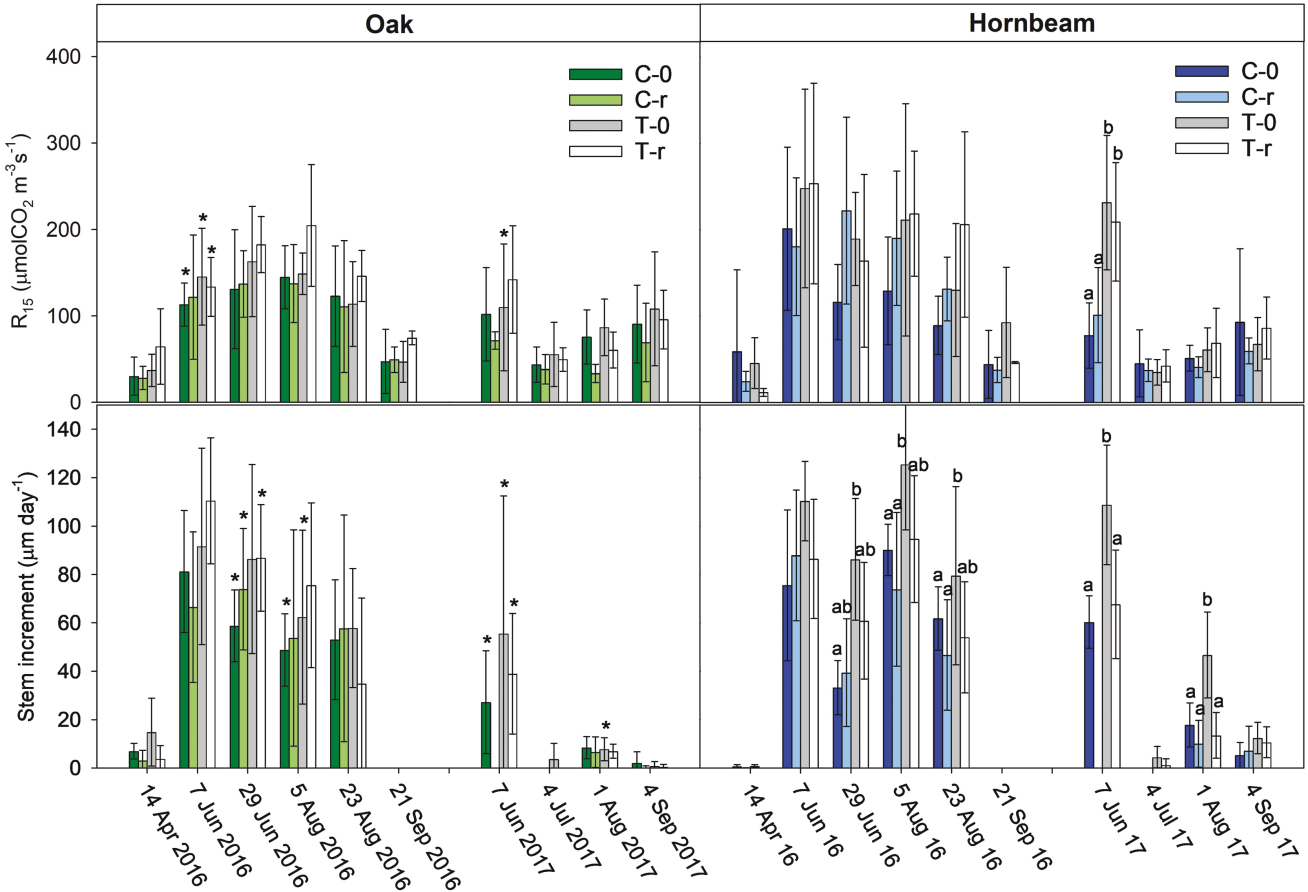
Mean (± SD) stem CO_2_ efflux at 15 °C (R_15_), and 7-day average of sprout diameter growth rate calculated from 5 to 6 sprouts per species and plot. Four plots with different treatments were studied: C-0, a coppice stand; T-0, a thinned coppice stand; C-r, a coppice stand with reduced throughfall and T-r, thinned coppice stand with reduced throughfall. The asterisks indicate statistically significant differences between oak and hornbeam and the different letters indicate statistically significant differences between the treatments on a particular date (*P* < 0.05; two-way ANOVA on repeated measures).

On 29 June 2016, the sprout increment rate of oak tended to be significantly higher than that of hornbeam, while we found the opposite trend on 5 August 2016 and 7 June 2017 (Fig. 3). The mixed model (Table 1) did not confirm an effect of throughfall reduction on stem increment. Thinning significantly increased the stem increment for hornbeam and the effect of the treatment combination was not significant. Some more detailed comparisons on the individual measurement days are in Fig. 3.

**Table 1.** Estimates of the effect of throughfall reduction, thinning and their combination on R_15_ and stem increment for oak and hornbeam.

Variable	Fixed effect	Variance of random effects		*P* value
		Tree	Measurement day	
R_15_				
Oak				
Intercept	90.01	673	1881	6.75 × 10^−5^
Thinning	11.42	–	–	0.614
Reduction	−10.35	–	–	0.467
Thinning:Reduction	24.28	–	–	0.283
Residual	–	1195		–
Hornbeam				
Intercept	90.72	541.6	4380	2.19 × 10−^3^
Thinning	40.56	–	–	0.03528*
Reduction	11.91	–	–	0.53158
Thinning:Reduction	−11.29	–	–	0.67584
Residual	–	4078		–
Stem increment				
Oak				
Intercept	28.6641	167.2	1151.3	3.69 × 10^−2^
Thinning	9.4319	–	–	0.2959
Reduct	0.8945	–	–	0.9242
Thinning:Reduct	−3.1971	–	–	0.8048
Residual	–	409.7		–
Hornbeam				
Intercept	34.481	101.4	1533.7	2.37 × 10^−2^
Thinning	22.989	–	–	0.0025*
Reduct	−2.114	–	–	0.7646
Thinning:Reduct	−16.470	–	–	0.1084
Residual		409.7		

The magnitude of TWD was generally lower for hornbeam than for oak. Maximum daily TWD was low in April and May in both years and increased in the middle of June (Fig. 1). In 2016, it returned to low values and increased rapidly in September. In contrast, it persisted over the following months in 2017 and reached up to 360 (±108) µm for oak and 88 (±22) µm for hornbeam. The cumulative daily TWD maxima from July forward was higher in 2017 compared to 2016 [see Supporting Information Fig. S6] indicating higher water stress of the sprouts in 2017. This corresponds with the lower stem increment in 2017 (Fig. 1).

### Stem CO_2_ efflux (Rs)

Rs ranged between 6 and 730 µmolCO_2_ m^–3^s^–1^ (per volume of wood), which corresponded to Rs between 0.06 and 5.79 µmolCO_2_ m^–2^s^–1^ (per surface area of stem). The maximum rates occurred during the summer 2016. To eliminate the effect of changes in temperature during the measurement campaigns on Rs, we normalized Rs to 15 °C (R_15_). Similarly to Rs, R_15_ was the highest in the summer of 2016 when the mean values ranged between 113 and 205 µmolCO_2_ m^–3^s^–1^ for oak and between 116 and 254 µmolCO_2_ m^–3^s^–1^ for hornbeam (Fig. 3).

R_15_ was highly variable among the sprouts, but we did not find any significant relationship with the sprout diameter or 7-day average diameter increment of the individual sprouts on any of the measurement dates, therefore the sprout size did not explain the spatial variability in R_15_. R_15_ of oak and hornbeam sprouts was similar over both growing seasons. Significant differences between the species were confirmed only on 7 June 2016 and 7 June 2017 (Fig. 3). The mixed model did not confirm any effect of throughfall reduction on R_15_ (Table 1). However, R_15_ in the summer of 2016 was substantially higher than in 2017, which was characterized by continuously low SWC and low stem increment.

**Figure 4.**
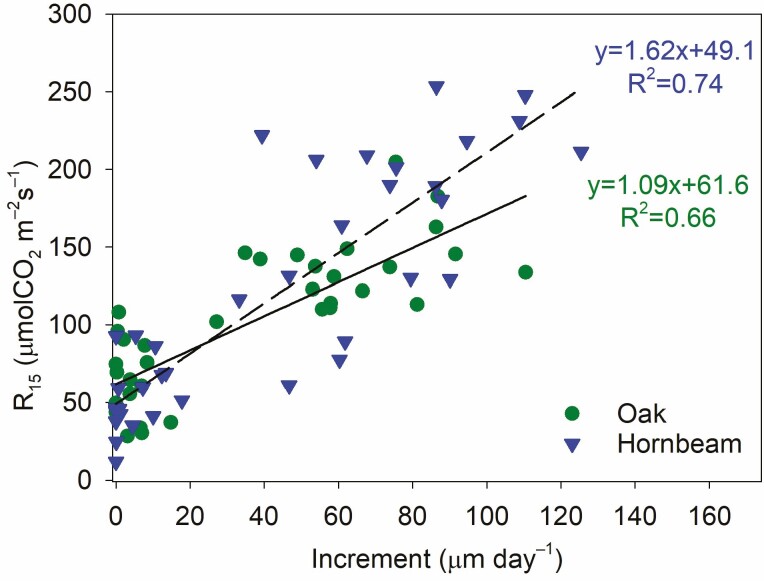
R_15_ over mean increment rate for oak and hornbeam from all treatments together. Dots show means calculated from five to six individuals for each measurement campaign in the growing seasons of 2016 and 2017.

The seasonal changes in R_15_ significantly correlated (*P* < 0.0001) with the mean stem increment rate through both growing seasons (Fig. 4). R_15_ under zero increment was, according to ANCOVA significantly (*P* < 0.05) higher for oak compared to hornbeam. On the other hand, the linear regression between R_15_ and stem increment rate was significantly (*P* < 0.05) steeper for hornbeam, showing higher R_15_ for hornbeam than oak above the growth rates of 30 µm day^–1^. At the increment rate of 100 µm m^–3^, which was approximately the maximum increment rate for both species, R_15_ amounted to 280 and 430 % of R_15_ at the zero increment rate for oak and hornbeam, respectively.

**Figure 5.**
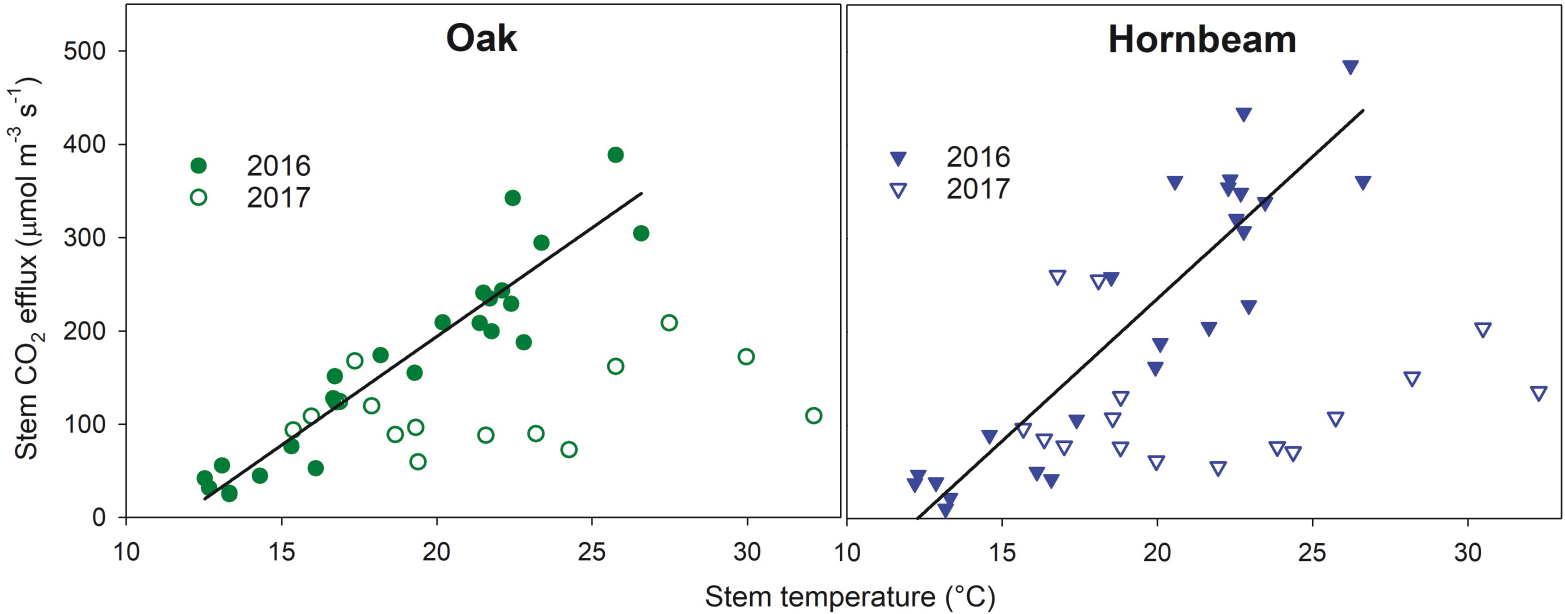
Stem CO_2_ efflux over stem temperature during two growing seasons (2016 and 2017) and liner regressions for 2016, a year with no severe drought. Each dot represents the mean value from each plot and date. The parameters of the linear regressions are summarized in Table 2.

To assess the effect of the summer drought in 2017, we analysed the relationship between Rs and temperature. Rs in 2016 increased with stem temperature and the relationship was best characterized with the linear regression. The parameters of the linear regressions between Rs and stem temperature for each tree species are summarized in Table 2. In 2017, Rs on 7 June and 4 September were close to the linear fit from 2016, but Rs on 4 July and 4 August was much lower than expected from the linear fits (Fig. 5). This corresponds with the period of a lower sprout diameter increment rate compared to 2016 (Fig. 3) due to the low water availability since mid-June 2017 (Fig. 1). During this period of 2017, Rs only reached 23–57 % and 19–37 % of Rs expected from the actual stem temperature and linear regressions from 2016 for oak and hornbeam, respectively (Table 2). The proportional decrease in Rs was generally bigger for hornbeam than for oak.

**Table 2.** Parameters of the linear regressions between stem temperature and stem CO_2_ efflux (Rs) in 2016, mean (±SD) residuals of Rs measured in 2017 from these regressions and mean (±SD) relative proportion of Rs measured in 2017 to Rs estimated on the basis of the linear regressions. The means were calculated from four plots for each date.

	Oak	Hornbeam
2016—linear regression		
Slope	23.3	30.5
Intercept	−271.5	−374.2
*R*^2^	0.89	0.83
2017—Rs residuals (µmol m^−3^s^−1^)		
7 June	−8.3 (±50.8)	23.2 (±91.9)
4 July	−165.7 (±43.7)	−246.3 (±54.7)
1 August	−237.1 (±97.3)	−365.2 (±75.4)
4 September	−22.4 (±37.5)	−70 (±47.9)
2017—%		
7 June	98.3 (±31.0)	116.8 (±60.5)
4 July	32.4 (±5.5)	21.6 (±3.4)
1 August	42.3 (±14.6)	29.2 (±6.2)
4 September	86.9 (±24.3)	60.4 (±23.0)

During September, the differences in Rs between 2016 and 2017 decreased (Table 2), especially for oak, despite persisting drought. However, there was negligible growth activity of the sprouts during the September campaigns in both years (Fig. 3).

## Discussion

This experiment was designed to investigate the effect of throughfall reduction on coppiced oak and hornbeam. Lower SWC at a depth of 25 cm was expected under the throughfall reduction treatment compared to the untreated plots. However, this was observed only for the unthinned coppice stands, while the opposite trend was observed for the thinned stands (Fig. 2). SWC is a spatially heterogeneous parameter (Darenova and Čater 2020) and our one-point measurements may not have been representative of entire plots. Moreover, the varied observations of soil rock fragments may reduce the absolute values of SWCs. Darenova *et al.* (2018b) measured SWC in the profile of 0–6 cm on the same plots during several campaigns in 2015 and 2016 and observed a slightly higher SWC on T-0 compared to C-0 and lower SWC on both throughfall reduction plots (up to about 5 %). A positive effect of thinning on soil moisture was also confirmed by Fedorová *et al.* (2018) at depths of 5, 10, 20, 30 and 40 cm on the same plots in 2015 as well as by del Campo *et al.* (2019) in a holm oak coppice forest.

Different soil water availability was observed between the growing seasons in 2016 and 2017 despite the similar amount of precipitation. We observed lower SWC in 2017 than in 2016 (Fig. 1) which can be assumed to be a result of higher temperature and consequent higher VPD [see Supporting Information Fig. S3]. High VPD is connected with higher losses of water through transpiration (Will *et al.* 2013), which can lead to the depletion of water in the soil (Zhu *et al.* 2024). We assume that both lower SWC and higher VPD in 2017 led to a higher TWD compared to 2016, which was observed in July and August. A substantial water shortage is reflected in reduced stem increment (Salomón *et al.* 2022), which corresponds to our results as we observed only about a half-annual increment in 2017 compared to 2016 [see Supporting Information Fig. S5]. The differences in TWD between the species can be explained by differences in below and aboveground biomass (Szatniewska *et al.* 2022). Moreover, sessile oak is classified as a strongly anisohydric species (Pretzsch *et al.* 2013), which means it keeps stomata open during drought. In contrast, hornbeam exhibits more isohydric behaviour (Leuschner *et al.* 2019) when trees prevent water losses by closing their stomata. Higher TWD for oak compared to hornbeam was observed also by Szatniewska *et al*. (2022).

Stem CO_2_ efflux (Rs) reflects the CO_2_ production from the respiration of living cells. There is a discrepancy between these two processes (Teskey *et al.* 2008) that can be caused by a portion of CO_2_ getting dissolved in sap and being transported upward in the xylem stream. Therefore, the Rs rate is a few percent lower than the rate of respiration during the daytime on the same stem segment. Moreover, stem respiration during the day can be attenuated due to a lowered turgor of living cells (Saveyn *et al.* 2007b). Thus, daytime measurements of stem CO_2_ efflux, like in our experiment, generally underestimate daily stem respiration estimates (Salomón *et al.* 2018).

During day time, the trees are able to reduce the CO_2_ emission from their stems by refixing some of the CO_2_ diffusing out through corticular photosynthesis (Pfanz *et al.* 2002). Its intensity is strongly affected by the light transmitted through the canopy to the measured stem segment (Tarvainen *et al.* 2018). In our plots, the light intensity close to the ground was low, reported canopy gap fraction less than 10 %. Moreover, we used opaque chambers. Therefore, we assumed the impact of the corticular photosynthesis on our measurements negligible Darenova *et al.* (2018b) decrease in light intensity within the forest stand and we, moreover, used opaque chambers.

The investigated CO_2_ efflux from the stems of oak and hornbeam coppices was driven by temperature and growth. During the year with a favourable precipitation regime, Rs had a distinct seasonal trend with maxima during the summer months. That is in accordance with other studies focussed on temperate forests (e.g. Maier *et al.* 2010; Han *et al.* 2017; Darenova *et al.* 2018a; Zhao *et al.* 2018) and it is a result of a seasonal pattern in temperature and stem growth (Figs. 2 and Fig. 3). This trend was dramatically different in 2017 when SWC substantially decreased in summer and remained at low levels for a long time.

Insufficient water availability may change the water status in stem cells and reduce stem growth, which leads to a decrease in stem CO_2_ efflux (Saveyn *et al.* 2007b). Similarly to Rodríguez-Calcerrada *et al.* (2011), the reduction of throughfall by about 30 % did not affect stem increment nor Rs. Measured stem increment includes only volumetric changes in stems, which are mainly attributed to water intake (Hilty *et al.* 2021). Meanwhile, water is also essential for the thickening of cell walls (Cresswell *et al.* 2021), which is not captured by dendrometers. However, our results suggest that neither of these growth processes was affected by throughfall reduction because we did not confirm its effect on stem increments or CO_2_ efflux.

R_15_ at the stem increment rate of 0 µm day^–1^ (Fig. 4) should correspond to CO_2_ efflux resulting from maintenance respiration at 10 °C and anything exceeding this level can be assumed to be connected with CO_2_ efflux resulting from growth respiration (Lavigne and Ryan 1997). Hornbeam showed a steeper increase in R_15_ with stem increment rate, which indicates higher respiration costs connected with stem growth processes. As mentioned above, the measured stem increment recorded only volumetric changes in stems. The thickening of cell walls requires a higher carbon supply from photosynthesis (Krejza *et al.* 2022) and, according to Darenova *et al.* (2020), is more energy-demanding than the volumetric increment. Oak and hornbeam are both hardwood species, but hornbeam has slightly higher wood and bark densities (Pásztory *et al.* 2014; Petráš *et al.* 2021). This corresponds to our results because it indicates that more material is deposited in cell walls and that more energy needs to be gained from respiration for material synthesis.

The relationship between Rs and temperature is characterized as exponential (Han *et al.* 2017; Chan *et al.* 2018; Darenova *et al.* 2018a). In our case, however, the linear fit better characterized the relationship between these two variables over the growing season of 2016. First, the linear regression had a higher *R*^2^ than the exponential regression. Second, the curvature of the exponential regression led to unrealistically high Rs above the temperature of 25 °C. Fitting Rs and temperature over the whole season also includes the effect of growth that is highest in summer as along with temperature. Thus, the exponentiality of the Rs–temperature relationship might be biased. Darenova *et al.* (2018a) showed that separating the year or growing season into shorter periods with similar growth rates is more suitable for estimating the Rs dependence on temperature. We did not have enough data for this tactic. However, we assume that our approach is suitable for the estimation of the effect of the drought in 2017 on Rs. We assessed the dependence behaviour of Rs on temperature during the growing season between 12 °C and 27 °C; we found that Rs in July and August decreased to 19 %–57 % of Rs expected from the 2016 data. During the summer of 2017, Rs of both species decreased at similarly low rates (Fig. 3). Therefore, the bigger difference in Rs between 2016 and 2017 for hornbeam can be associated with its steeper relationship between Rs and temperature and between R_15_ and stem increment compared to oak. Both temperature and stem increment in 2016 had their maxima during summer. In contrast to our research, Rodríguez-Calcerrada *et al.* (2014) used shoot water potential as an indicator of water availability and showed that Rs at 15 °C of *Quercus ilex* coppice decreased by about 50 % after the decrease in shoot water potential from about –0.3 to –3.0 (MPa). Salomón *et al.* (2016) did not confirm any significant reduction of Rs in sub-Mediterranean oak (a coppice stand transformed to a mature forest) below the shoot water potential of −1.4 MPa. That suggests that such a level of drought does not yet affect Rs.

In contrast to the notable impact of drought in June and August, the effect of the persisting drought diminished in September when a negligible stem increment activity was recorded in both 2016 and 2017. Despite zero stem increment during the September campaigns, we can assume that other stem growth processes, such as cell wall thickening, were still ongoing during that time in 2016 as they have a delay behind the stem volumetric changes recorded by dendrometers (Darenova *et al.* 2020). This can explain the persisting differences in Rs in September. Nevertheless, the results indicate that not only the magnitude but also the timing of drought is crucial in Rs response. This agrees with the observation from Krejza *et al.* (2022) that drought from May to June affected negatively annual stem increments of Norway spruce in a temperate area where this period corresponds to the onset and expansion of the stem increment. Conversely, D’Andrea *et al.* (2020) observed a negligible effect of drought, which occurred at the end of the growing season, on Rs. Moreover, the whole growing season can be negatively affected by the drought during the pre-growing season (Wu *et al.* 2022).

Thinning is a common practice to improve the development of trees and mitigate the impact of climate change on coppiced trees. For example, reducing stand density delays the date of drought-induced growth cessation (Cabon *et al.* 2018). Thinning reduces the competition among sprouts and leads to an increase in stem increment rate as was also shown in a similar study by Rodríguez-Calcerrada *et al*. (2011). Thinning tended to increase increment rate during the high growing season, especially for T-0 of hornbeam. However, the results of this study were likely not as clear as in the study of Rodríguez-Calcerrada *et al*. (2011) because of the high variability among the individual sprouts. Nevertheless, a significantly higher stem increment was observed in the first year (2015) after the treatment in both species by Fedorová *et al.* (2018). Moreover, the summer drought in 2017 severely limited the coppice growth regardless of treatment. Higher increment rates in thinned plots did not reflect in Rs; statistically significant differences among treatments were confirmed only on 7 June 2017.

To fully understand the effect of thinning, throughfall reduction, and natural water availability fluctuation on coppice stem growth as one of the main drivers of Rs, more detailed research of a longer and continuous period will be performed in the future.

## Conclusions

Our results demonstrate that a mild precipitation reduction has no effect on stem increment and stem CO_2_ efflux of oak and hornbeam coppices. The severe drought, which occurred between July and September 2017, substantially decreased stem CO_2_ efflux by 17–55 % in July and August and thinned coppices did not show any improvement in the response to the drought. During this period, the stem increment rate was also severely reduced. Although the drought persisted until September, stem CO_2_ efflux differed very little compared to 2016 when SWC was favourable. During September of both years, stem growth activity was no longer observed. That leads to the conclusion that drought exclusively affected the growth respiration component of stem CO_2_ efflux. More measurements need to be taken during the non-growing season when stem CO_2_ efflux is a result of only maintenance respiration and after longer exposure to throughfall reductions to determine if or to what extent drought affects the maintenance respiration component. Nevertheless, this study showed that the expected increase in the frequency of summer droughts will substantially affect carbon source-sink relationships in the forests.

## Supporting Information

The following additional information is available in the online version of this article –

Figure S1. Mean (SD) diameters of the stem segments of oak and hornbeam sprouts enclosed in the respiration chamber.

Figure S2. A scheme of a chamber for stem CO_2_ efflux from the coppice sprouts.

Figure S3. Mean monthly air humidity and VPD in 2016 and 2017.

Figure S4. Monthly precipitation (a), mean air temperature (b) and mean air humidity (c) for the climate normal 1981–2010 and the experimental years 2016 and 2017. The data are obtained from the nearest long-term meteorological station Turany Brno (CHMI 2024) which is 10.5 km south of the experimental plots.

Figure S5. Mean cumulative stem diameter increment of oak and hornbeam sprouts in the control plot over two growing seasons.

Figure S6. Cumulative maxima of tree water deficit (TWD) of oak and hornbeam sprouts over two growing seasons.

plae023_suppl_Supplementary_Figures

## Data Availability

Data presented in this study are available on https://doi.org/10.57680/asep.0584705.

## References

[CIT0001] Acosta M , Pavelka M, Pokorný R, Janouš D, Marek MV. 2008. Seasonal variation in CO_2_ efflux of stems and branches of Norway spruce trees. Annals of Botany 101:469–477. 18057065 10.1093/aob/mcm304 PMC2701814

[CIT0002] Allen RG , Pereira LS, Raes D, Smith M. 1998. Crop evapotranspiration. FAO Irrigation and Drainage Paper No. 56. Rome: FAO.

[CIT0003] Amthor JS. 2000. The McCree-de Wit-Penning de Vries-Thornley respiration paradigms: 30 Years later. Annals of Botany 86:1–20.

[CIT0004] Babst F , Bouriaud O, Papale D, Gielen B, Janssens Ivan A, Nikinmaa E, Ibrom A, Wu J, Bernhofer C, Köstner B, et al. 2014. Above-ground woody carbon sequestration measured from tree rings is coherent with net ecosystem productivity at five eddy-covariance sites. New Phytologist 201:1289–1303. 24206564 10.1111/nph.12589

[CIT0005] Cabon A , Mouillot F, Lempereur M, Ourcival JM, Simioni G, Limousin JM. 2018. Thinning increases tree growth by delaying drought-induced growth cessation in a Mediterranean evergreen oak coppice. Forest Ecology and Management 409:333–342.

[CIT0006] Chan T , Berninger F, Kolari P, Nikinmaa E, Hölttä T. 2018. Linking stem growth respiration to the seasonal course of stem growth and GPP of Scots pine. Tree Physiology 38:1356–1370. 29771366 10.1093/treephys/tpy040 PMC6178967

[CIT0007] CHMI. 2024. Historical data—meteorology and climatology. Czech hydrometeorological institute.

[CIT0008] Cresswell R , Dupree R, Brown SP, Pereira CS, Skaf Munir S, Sorieul Mathias, Dupree P, Hill S. 2021. Importance of water in maintaining softwood secondary cell wall nanostructure. Biomacromolecules 22:4669–4680. 34669375 10.1021/acs.biomac.1c00937 PMC8579401

[CIT0009] D’Andrea E , Rezaie N, Prislan P, Gričar J, Collalti A, Muhr J, Matteucci G. 2020. Frost and drought: effects of extreme weather events on stem carbon dynamics in a Mediterranean beech forest. Plant Cell and Environment 43:2365–2379. 10.1111/pce.13858 32705694

[CIT0010] Darenova E , Čater M. 2020. Effect of spatial scale and harvest on heterogeneity of forest floor CO_2_ efflux in a sessile oak forest. Catena 188:104455.

[CIT0011] Darenova E , Acosta M, Pokorny R, Pavelka M. 2018a. Variability in temperature dependence of stem CO_2_ efflux from Norway spruce trees. Tree Physiology 38:1333–1344. 29425384 10.1093/treephys/tpy006

[CIT0012] Darenova E , Crabbe RA, Knott R, Uherková B, Kadavý J. 2018b. Effect of coppicing, thinning and throughfall reduction on soil water content and soil CO_2_ efflux in a sessile oak forest. Silva Fennica 52:9927.

[CIT0013] Darenova E , Horácek P, Krejza J, Pokorný R, Pavelka M. 2020. Seasonally varying relationship between stem respiration, increment and carbon allocation of Norway spruce trees. Tree Physiology 40:943–955. 32268373 10.1093/treephys/tpaa039

[CIT0014] del Campo AD , González-Sanchis M, García-Prats A, Ceacero CJ, Lull C. 2019. The impact of adaptive forest management on water fluxes and growth dynamics in a water-limited low-biomass oak coppice. Agricultural and Forest Meteorology 264:266–282.

[CIT0015] del Campo AD , Otsuki K, Serengil Y, Blanco JA, Yousefpour R, Wei X. 2022. A global synthesis on the effects of thinning on hydrological processes: implications for forest management. Forest Ecology and Management 519:120324.

[CIT0016] De Lucia EH , Drake JE, Thomas RB, Gonzalez-Meler M. 2007. Forest carbon use efficiency: is respiration a constant fraction of gross primary production? Global Change Biology 13:1157–1167.

[CIT0017] Evans J. 1992. Coppice forestry—an overview In: Buckley GP, ed. Ecology and management of coppice woodlands. Chapman & Hall, Springer Netherlands,18–27.

[CIT0018] Fedorová B , Kadavý J, Adamec Z, Knott R, Kučera A, Kneifl M, Drápela K, Inurrigarro RO. 2018. Effect of thinning and reduced throughfall in young coppice dominated by *Quercus petraea* (Matt.) Liebl. and *Carpinus betulus* L. Austrian Journal of Forest Science 135:1–17.

[CIT0019] Fernández-martínez M , Vicca S, Janssens I, Luyssaert S, Campioli M, Sardans J, Estiarte M, Peñuelas J. 2014. Spatial variability and controls over biomass stocks, carbon fluxes, and resource-use efficiencies across forest ecosystems. Trees—Structure and Function 28:597–611.

[CIT0020] Gruber A , Baumgartner D, Zimmermann J, Oberhuber W. 2009. Temporal dynamic of wood formation in *Pinus cembra* along the alpine treeline ecotone and the epaffect of climate variables. Trees—Structure and Function 23:623–635. 21509148 10.1007/s00468-008-0307-7 PMC3078619

[CIT0021] Han F , Wang X, Zhou H, Li Y, Hu D. 2017. Temporal dynamics and vertical variations in stem CO_2_ efflux of *Styphnolobium japonicum*. Journal of Plant Research 130:845–858. 28536983 10.1007/s10265-017-0951-3

[CIT0022] Herrero C , Juez L, Tejedor C, Pando V, Bravo F. 2014. Importance of root system in total biomass for *Eucalyptus globulus* in northern Spain. Biomass and Bioenergy 67:212–222.

[CIT0023] Hilty J , Muller B, Pantin F, Leuzinger S. 2021. Plant growth: the what, the how, and the why. The New Phytologist 232:25–41. 34245021 10.1111/nph.17610

[CIT0024] Jardine KJ , Tucker E, Dewhirst RA, Som S, Lei J, Young RP, Estrada MP, Su L, Fares S, Mortimer JC, et al. 2022. Cell wall ester modifications and volatile emission signatures of plant response to abiotic stress. Plant Cell and Environment 45:3429–3444. 10.1111/pce.14464 PMC9828120 36222152

[CIT0025] Kadavý J , Kneifl M, Knott R. 2011. Biodiversity and target management of endangered and protected species in coppices and coppices with standards included in system of Natura 2000: methodology of establishment of experimental research plots in the conversion to coppice and coppice-with-sta. Brno: Mendel University in Brno.

[CIT0026] Khomik M , Arain MA, Brodeur JJ, Peichl M, Restrepo-Coup N, McLaren JD. 2010. Relative contributions of soil, foliar, and woody tissue respiration to total ecosystem respiration in four pine forests of different ages. Journal of Geophysical Research, Biogeosciences 115:G03024.

[CIT0027] Krejza J , Haeni M, Darenova E, Foltýnová L, Fajstavr M, Světlík J, Nezval O, Bednář P, Šigut L, Horáček P, et al. 2022. Disentangling carbon uptake and allocation in the stems of a spruce forest. Environmental and Experimental Botany 196:104787.

[CIT0028] Kunert N , El-madany TS, Maria L, Aparecido T, Wolf S, Potvin C. 2019. Understanding the controls over forest carbon use efficiency on small spatial scales: effects of forest disturbance and tree diversity. Agricultural and Forest Meteorology 269–270:136–144.

[CIT0029] Lavigne MB , Ryan MG. 1997. Growth and maintenance respiration rates of aspen, black spruce and jack pine stems at northern and southern BOREAS sites. Tree Physiology 17:543–551. 14759827 10.1093/treephys/17.8-9.543

[CIT0030] Leuschner C , Wedde P, Lübbe T. 2019. The relation between pressure–volume curve traits and stomatal regulation of water potential in five temperate broadleaf tree species. Annals of Forest Science 76:60.

[CIT0031] Litschmann T. 1991. A soil moisture sensor and its application in agriculture. Communications in Soil Science and Plant Analysis 22:409–418.

[CIT0032] Liu C , Chen Z, Liu S, Cao K, Niu B, Liu X, Gao X. 2023. Multi-year throughfall reduction enhanced the growth and non-structural carbohydrate storage of roots at the expenses of above-ground growth in a warm-temperate natural oak forest. Forest Ecosystems 10:100118.

[CIT0071] Logli F , Joffre R. 2001. Individual variability as related to stand structure and soil condition in a Mediterranean oak coppice. *Forest Ecology and Management* 142:53–63.

[CIT0033] Maier CA , Johnsen KH, Clinton BD, Ludovici KH. 2010. Relationships between stem CO_2_ efflux, substrate supply, and growth in young loblolly pine trees. New Phytologist 185:502–513. 19878459 10.1111/j.1469-8137.2009.03063.x

[CIT0034] Malhi Y , Baldocchi DD, Jarvis PG. 1999. The carbon balance of tropical, temperate and boreal forests. Plant, Cell & Environment 22:715–740.

[CIT0035] Pásztory Z , Börcsök Z, Ronyecz I, Mohácsi K, Molnár S, Kis S. 2014. Oven dry density of sessile oak, Turkey oak and hornbeam in different region of Mecsek Mountain. Wood Research 59:683–694.

[CIT0036] Petráš R , Mecko J, Krupová D, Pažitný A. 2021. Aboveground biomass basic density of hardwood tree species. Wood Research 65:1001–1011.

[CIT0037] Pfanz H , Aschan G, Langenfeld-Heyser R, Wittmann C, Loose M. 2002. Ecology and ecophysiology of tree stems: corticular and wood photosynthesis. Naturwissenschaften 89:147–162. 12061398 10.1007/s00114-002-0309-z

[CIT0038] Pietras J , Stojanović M, Knott R, Pokorný R. 2016. Oak sprouts grow better than seedlings under drought stress. iForest 9:529–535.

[CIT0039] Pretzsch H , Schütze G, Uhl E. 2013. Resistance of European tree species to drought stress in mixed versus pure forests: evidence of stress release by inter-specific facilitation. Plant Biology (Stuttgart, Germany) 15:483–495. 23062025 10.1111/j.1438-8677.2012.00670.x

[CIT0040] Rambal S , Lempereur M, Limousin JM, Martin-Stpaul NK, Ourcival JM, Rodríguez-Calcerrada J. 2014. How drought severity constrains gross primary production (GPP) and its partitioning among carbon pools in a *Quercus ilex* coppice? Biogeosciences 11:6855–6869.

[CIT0041] Rodríguez-calcerrada J , Salomón RL, Gordaliza GG, Miranda JC, Miranda E, de Riva EG, Gil L. 2019. Respiratory costs of producing and maintaining stem biomass in eight co-occurring tree species. Tree Physiology 39:1838–1854. 31211374 10.1093/treephys/tpz069

[CIT0042] Rodríguez-Calcerrada J , Pérez-Ramos IM, Ourcival JM, Limousin JM, Joffre R, Rambal S. 2011. Is selective thinning an adequate practice for adapting *Quercus ilex* coppices to climate change? Annals of Forest Science 68:575–585.

[CIT0043] Rodríguez-Calcerrada J , Martin-StPaul NK, Lempereur M, Ourcival JM, Del Rey MC, Joffre R, Rambal S. 2014. Stem CO_2_ efflux and its contribution to ecosystem CO_2_ efflux decrease with drought in a Mediterranean forest stand. Agricultural and Forest Meteorology 195–196:61–72.

[CIT0044] Rodríguez-Calcerrada J , Rodrigues AM, António C, Perdiguero P, Pita P, Collada C, Li M, Gil L. 2021. Stem metabolism under drought stress—a paradox of increasing respiratory substrates and decreasing respiratory rates. Physiologia Plantarum 172:391–404. 32671841 10.1111/ppl.13145

[CIT0045] Rowland L , da Costa ACL, Oliveira AAR, Oliveira RS, Bittencourt PL, Costa PB, Giles AL, Sosa AI, Coughlin I, Godlee JL, et al. 2018. Drought stress and tree size determine stem CO_2_ efflux in a tropical forest. The New Phytologist 218:1393–1405. 29397028 10.1111/nph.15024 PMC5969101

[CIT0046] Salomón RL , Valbuena-Carabaña M, Gil L, McGuire MA, Teskey RO, Aubrey DP, González-Doncel I, Rodríguez-Calcerrada J. 2016. Temporal and spatial patterns of internal and external stem CO_2_ fluxes in a sub-Mediterranean oak. Tree Physiology 36:1409–1421. 27126229 10.1093/treephys/tpw029

[CIT0047] Salomón RL , Rodríguez-Calcerrada J, Staudt M. 2017. Carbon losses from respiration and emission of volatile organic compounds—the overlooked side of tree carbon budgets In: Gil-Pelegrín E, Peguero-Pina J, Sancho-Knapik D, eds. Oaks physiological ecology. Exploring the functional diversity of genus Quercus L. Tree Physiology, vol. 7. Cham: Springer, 327–359.

[CIT0048] Salomón RL , De Schepper V, Valbuena-Carabaña M, Gil L, Steppe K. 2018. Daytime depression in temperature-normalised stem CO_2_ efflux in young poplar trees is dominated by low turgor pressure rather than by internal transport of respired CO2. The New Phytologist 217:586–598. 28984360 10.1111/nph.14831

[CIT0049] Salomón RL , Peters RL, Zweifel R, Sass-Klaassen UGW, Stegehuis AI, Smiljanic M, Poyatos R, Babst F, Cienciala E, Fonti P, et al. 2022. The 2018 European heatwave led to stem dehydration but not to consistent growth reductions in forests. Nature Communications 13:28. 10.1038/s41467-021-27579-9 PMC8748979 35013178

[CIT0050] Saveyn A , Steppe K, Lemeur R. 2007a. Drought and the diurnal patterns of stem CO_2_ efflux and xylem CO_2_ concentration in young oak (*Quercus robur*). Tree Physiology 27:365–374. 17241978 10.1093/treephys/27.3.365

[CIT0051] Saveyn A , Steppe K, Lemeur R. 2007b. Daytime depression in tree stem CO_2_ efflux rates: is it caused by low stem turgor pressure? Annals of Botany 99:477–485. 17204535 10.1093/aob/mcl268 PMC2802950

[CIT0052] Schindlbacher A , Wunderlich S, Borken W, Kitzler B, Zechmeister-Boltenstern S, Jandl R. 2012. Soil respiration under climate change: prolonged summer drought offsets soil warming effects. Global Change Biology 18:2270–2279.

[CIT0053] Sieferle RP. 2001. The subterranean forest: energy systems and the industrial revolution. Cambridge: White Horse Press.

[CIT0054] Sousa VB , Cardoso S, Pereira H. 2013. Ring width variation and heartwood development in *Quercus faginea*. Wood and Fiber Science 45:405–414.

[CIT0055] Szatniewska J , Zavadilova I, Nezval O, Krejza J, Petrik P, Čater M, Stojanović M. 2022. Species-specific growth and transpiration response to changing environmental conditions in floodplain forest. Forest Ecology and Management 516:120248.

[CIT0056] Tarvainen L , Wallin G, Lim H, Linder S, Oren R, Löfvenius MO, Räntfors M, Tor-Ngern P, Marshall J. 2018. Photosynthetic refixation varies along the stem and reduces CO_2_ efflux in mature boreal *Pinus sylvestris* trees. Tree Physiology 38:558–569. 29077969 10.1093/treephys/tpx130

[CIT0057] Teskey RO , Saveyn A, Steppe K, McGuire MA. 2008. Origin, fate and significance of CO_2_ in tree stems. The New Phytologist 177:17–32. 18028298 10.1111/j.1469-8137.2007.02286.x

[CIT0058] Van Kampen R , Fisichelli N, Zhang YJ, Wason J. 2022. Drought timing and species growth phenology determine intra-annual recovery of tree height and diameter growth. AoB Plants 14:plac012. 35558163 10.1093/aobpla/plac012 PMC9089829

[CIT0059] West PW. 2020. Do increasing respiratory costs explain the decline with age of forest growth rate? Journal of Forestry Research 31:693–712.

[CIT0060] Will RE , Wilson SM, Zou CB, Hennessey TC. 2013. Increased vapor pressure deficit due to higher temperature leads to greater transpiration and faster mortality during drought for tree seedlings common to the forest-grassland ecotone. The New Phytologist 200:366–374. 23718199 10.1111/nph.12321

[CIT0061] Wolf S , Paul-Limoges E. 2023. Drought and heat reduce forest carbon uptake. Nature Communications 14:14–17. 10.1038/s41467-023-41854-x PMC10558509 37802990

[CIT0062] Wu XC , Liu HY, Hartmann H, Ciais P, Kimball JS, Schwalm CR, Camarero JJ, Chen AP, Gentine P, Yang YT, et al. 2022. Timing and order of extreme drought and wetness setermine bioclimatic sensitivity of tree growth. Earth’s Future 10:e2021EF002530.

[CIT0063] Xiao JL , Zeng F, He QL, Yao YX, Han X, Shi WY. 2021. Responses of forest carbon cycle to drought and elevated CO_2_. Atmosphere 12:212.

[CIT0064] Yang J , He Y, Aubrey DP, Zhuang Q, Teskey RO. 2016. Global patterns and predictors of stem CO_2_ efflux in forest ecosystems. Global Change Biology 22:1433–1444. 26667780 10.1111/gcb.13188

[CIT0065] Zha T , Kellomäki S, Wang KY, Ryyppö A, Niinistö S. 2004. Seasonal and annual stem respiration of scots pine trees under boreal conditions. Annals of Botany 94:889–896. 15469943 10.1093/aob/mch218 PMC4242285

[CIT0066] Zhao K , Zheng M, Fahey TJ, Jia Z, Ma L. 2018. Vertical gradients and seasonal variations in the stem CO_2_ efflux of *Larix principis-rupprechtii* Mayr. Agricultural and Forest Meteorology 262:71–80.

[CIT0067] Zhou S , Park Williams A, Berg AM, Cook BI, Zhang Y, Hagemann S, Lorenz R, Seneviratne SI, Gentine P. 2019. Land–atmosphere feedbacks exacerbate concurrent soil drought and atmospheric aridity. Proceedings of the National Academy of Sciences of the United States of America 116:18848–18853. 31481606 10.1073/pnas.1904955116 PMC6754607

[CIT0068] Zhu XL , Si JH, He XH, Jia B, Zhou DM, Wang CL, Qin J, Liu ZJ. 2024. Effects of long-term afforestation on soil water and carbon in the Alxa plateau. Frontiers in Plant Science 14:1273108. 38273949 10.3389/fpls.2023.1273108 PMC10808672

[CIT0069] Zweifel R , Haeni M, Buchmann N, Eugster W. 2016. Are trees able to grow in periods of stem shrinkage? The New Phytologist 211:839–849. 27189708 10.1111/nph.13995

[CIT0070] Zweifel R , Sterck F, Braun S, Buchmann N, Eugster W, Gessler A, Häni M, Peters RL, Walthert L, Wilhelm M, et al. 2021. Why trees grow at night. The New Phytologist 231:2174–2185. 34118158 10.1111/nph.17552 PMC8457160

