# Supplementary material for: Does lower water availability limit stem CO2 efflux of oak and hornbeam coppices?
Source: AoB Plants. 2024 Apr 5;16(2):plae023. doi: 10.1093/aobpla/plae023 (PMC11025467; doi:10.1093/aobpla/plae023)
Supplement: plae023_suppl_Supplementary_Figures [file plae023_suppl_supplementary_figures.pdf]

Supplementary information

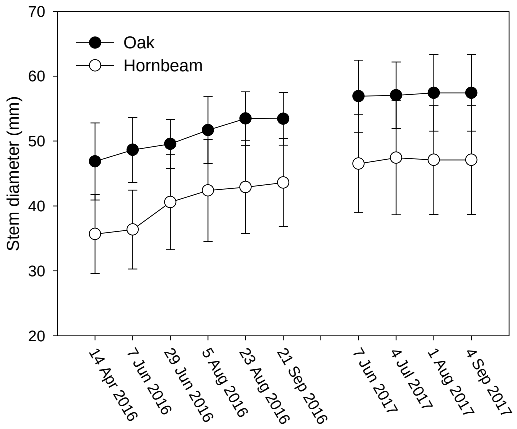

Figure S1. Mean (SD) diameters of the stem segments of oak and hornbeam sprouts enclosed in the respiration chamber.

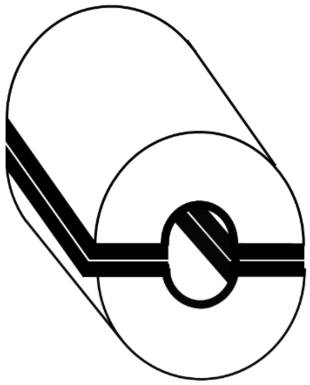

Figure S2. A scheme of a chamber for stem CO_2 efflux from the coppice sprouts.

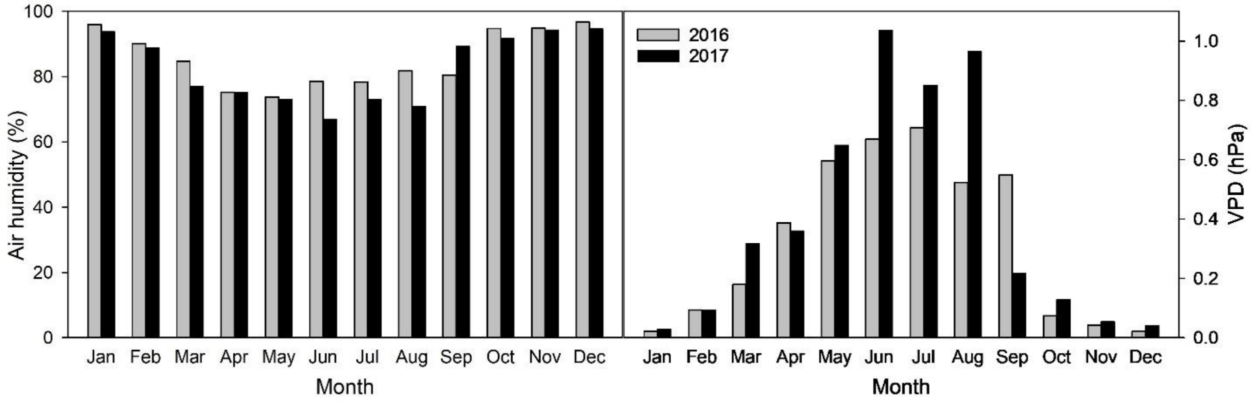

Figure S3. Mean monthly air humidity and vapour pressure deficit (VPD) in 2016 and 2017.

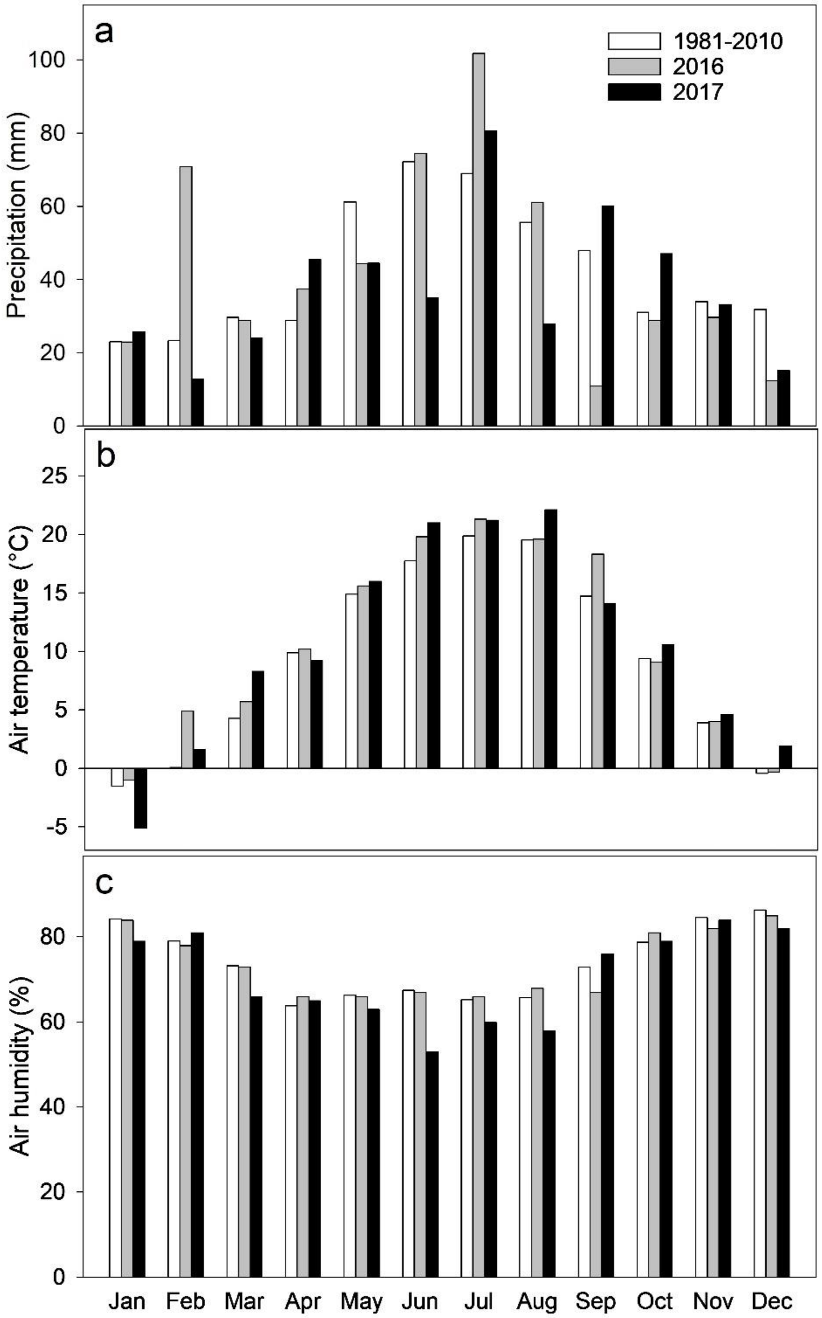

Figure S4. Monthly precipitation (a), mean air temperature (b), and mean air humidity (c) for the climate normal 1981-2010 and the experimental years 2016 and 2017. The data are obtained from the nearest long-term meteorological station Turany Brno (CHMI 2024) which is 10.5 km south of the experimental plots.

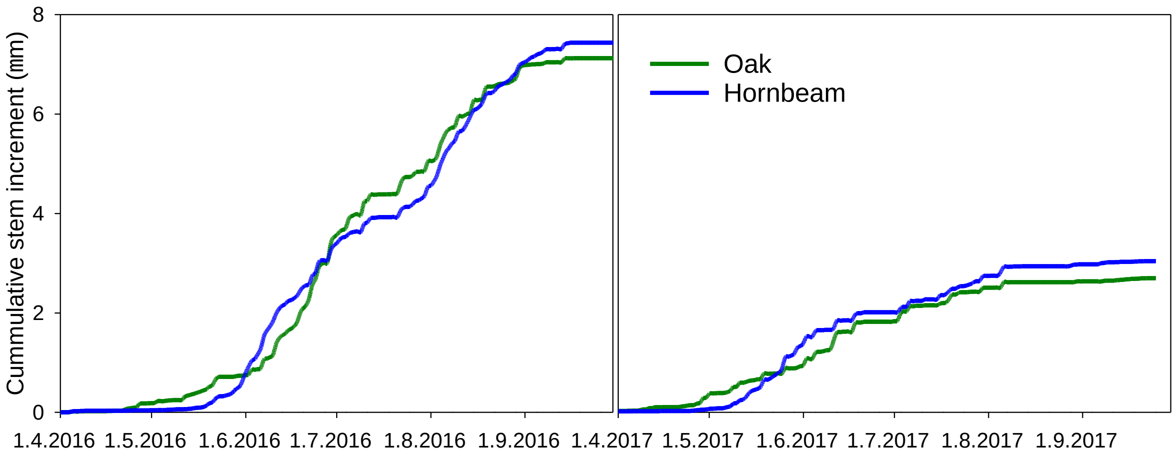

Figure S5. Mean cumulative stem diameter increment of oak and hornbeam sprouts in the control plot over two growing seasons.

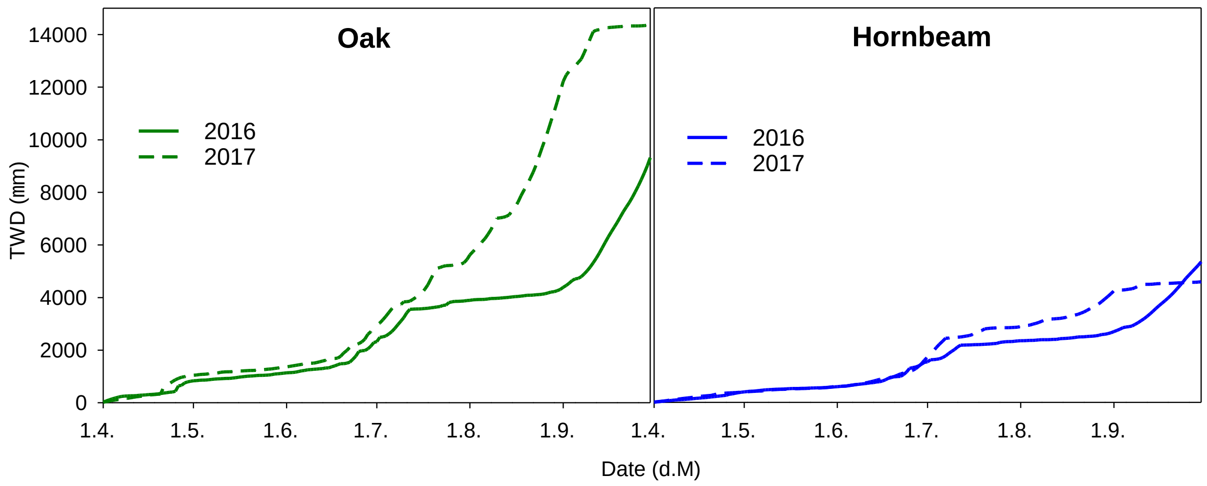

Figure S6. Cumulative maxima of tree water deficit (TWD) of oak and hornbeam sprouts over two growing seasons.
